# Exploring The Future of Prosthetics and Orthotics: Harnessing The Potential of 3D Printing

**DOI:** 10.33137/cpoj.v6i2.42140

**Published:** 2023-12-22

**Authors:** A.R Gutierrez

**Affiliations:** Bionic Prosthetics and Orthotics Group LLC, Merrillville, Indiana, USA.

**Keywords:** Orthotics, Prosthetics, 3D Printing, Additive Manufacturing, Digital Workflow, Diagnostic Sockets, Material Strength, Clinical Efficiency, Prosthetic Interface

## Abstract

This paper explores the transformative impact of 3D printing on Orthotics and Prosthetics, focusing on enhancing patient outcomes and clinical efficiency. Over the past decade, the integration of additive manufacturing has revolutionized device fabrication, particularly in diagnostic socket production, leading to significant time reductions in patient care. This article addresses challenges such as material limitations and the need for equivalent strength to traditional sockets, exploring the use of PETG filaments and advanced printers. It emphasizes the role of digital scanning and model modification technology, highlighting affordable solutions like Structure Sensor Scanners and iPhone-based capture systems in shaping the digital workflow. The importance of a standardized digital workflow in clinical settings is discussed, showcasing reduced practitioner time and improved patient care. The paper concludes by outlining ongoing efforts to enhance patient care through automation and flexible prints. In summary, this paper provides a concise overview of the impactful advancements in Orthotics and Prosthetics through 3D printing, highlighting its potential for improved clinical efficiency and patient outcomes.

## INTRODUCTION

In the last decade, Prosthetics and Orthotics (P&O) have embraced technological progress, particularly in bionic advancements, boosting patient mobility outcomes. Recently, focus has turned to enhancing the P&O interface, crucial for effective clinical interventions. Over the past 6+ years, 3D printing has transformed accessibility, workflow, and implementation, impacting prosthetic and orthotic procedures. 3D printing in general, is a robust tool for fabricating complicated objects in a cost-effective and timely manner.^1^ Despite initial challenges, collaborative efforts between technical printer manufacturers, software developers, and clinicians have streamlined digital workflows, promoting increased adoption of this technology in clinical practices.

Our clinical group, Bionic Prosthetics and Orthotics Group (www.bionicpo.com), has been at the forefront of implementation of 3D printing into our clinical practice, primarily in Prosthetic cases, utilizing FDM (Fused Deposition Modeling).

We were attempting to standardize and improve the timeliness of our clinical workflow and overcome the traditional time-consuming fabrication methods of hand casting, plaster model modifications, thermoforming and lamination to enable our clinicians to spend more time focusing on patient care. Rapid production in 3D printed sockets may shorten the time from evaluation to delivery of the prosthesis,^2^ socket modifications after limb shape changes; which could improve clinical outcomes in prosthesis use^3^ and limit any negative effects of socket disuse.^4^ For this transformation to occur, we had to demonstrate outcomes of printed devices could match those attained with standard fabrication devices. The key challenges to penetrating this space in our practice were digital scanning technology, printing materials and strength, printing time, and digital workflow efficiency.

## DIGITAL SHAPE CAPTURE

During this period, the P&O field had access to CAD/CAM technology with white light scanners, offering heightened accuracy. Yet, the integration across multiple clinic locations was challenging due to financial, hardware, and reliability limitations. Despite having a few white light scanners within our practice, the consistency and efficiency were burdensome, often exceeding the time of traditional methods; additionally, lacking an in-house carver, we resorted to costlier central fabrication for diagnostic sockets.

A pivotal shift occurred when Structure Sensor Scanners, coupled with an Apple iPad, entered the scene, providing affordable scanning. This enabled our practitioners to do quick limb scanning with a familiar technology, a crucial efficiency lesson for our journey towards standardized 3D printing integration.

The importance of consistent shape capture across a large team is vital to the program's success. Our group has traditionally used the Structure Sensor and iPad interface, but recently the availability of new Structure Sensors has been limited and has created a challenge as our technology is aging and accuracy issues were realized. We have recently transitioned to Orten capture software (Orten 3D Cam 6.0.1 (588)) (https://orten.proteor.com/orten), utilizing the iPhone front camera system. All clinicians on our team utilize the same phone for clinical purposes, and this was an ideal solution to create uniformity across all scans coming into the fabrication team, which has led to more consistent output.

## MATERIAL LIMITATIONS

At the time that we started this work, PLA (Polylactic Acid) and ABS (Acrylonitrile Butadiene Styrene) were plastic filaments that were widely available and utilized by hobby printers and were the first materials utilized in the P&O space. While these filaments are easy to print with, the material was not familiar to the P&O clinician to utilize in a clinical sense. In the prosthetic clinical workflow, a vital part of the process is the diagnostic socket fitting. These sockets are utilized as a platform to create the definitive fabrication of the prosthesis. Clinicians are often modifying the socket shape via heat and removal of material to create the customized socket that will be implemented as a long-term solution for the patient. The problem with printed sockets was that PLA and ABS do not behave like traditional thermoplastic materials used by the field in the modification process necessary during diagnostic fittings, thus our clinicians struggled with implementing 3D printed solutions into their practice. In time, we were able to find more suitable PETG filaments that could be utilized and manipulated with similar methods to traditional fabrication. However, our initial printers were standard flow FDM printers (with a nozzle dimension of .6mm utilizing 1.25 mm filament), and in order to print a diagnostic socket which was strong enough withstand socket donning and static weight bearing, we needed to print a socket with sufficient material thickness to ensure strength during weight-bearing and dynamic alignment of the prosthesis. To accomplish this, we had to print with an inner shell, an internal honeycomb structure, and an outer shell to complete the socket shape. While this was able to create a socket that was strong enough for fittings, it was opaque, and thus not an equivalent process to traditional fabrication as modification of the material was still a challenge due to the dual wall design with 25–50% infill based on patient activity characteristics.

## PRINTING TIME

In our traditional workflow, a limb impression is taken, filled with plaster, hand modified, thermoformed, and trimmed. This process would involve about 1 hour of practitioner time and 1.5 hours of technician time. In our initial digital workflow, with a standard flow printer, we could capture the limb impression and digitally modify it in about 15 minutes, then the socket could be designed for printing in about 10 minutes. However, depending on the size of the residual limb, it takes about 8–12 hours to 3D print a PETG or Nylon socket with 25–50% infill percentage using a Filament Innovations ICARUS GEN 1.0 Printer. While practitioner time was greatly reduced, we were still behind on the technical timeline and still had not created a more efficient process, as we had one standard flow printer servicing nine clinics. The first breakthrough of printed sockets in mass adaptation into our workflow was when we partnered with Filament Innovations and their High-Flow Kratos, which was able to utilize a 2.5 mm nozzle and 2.85 mm filament. The output of this machine gave us the closest replica of a standard fabrication diagnostic socket. The printer was able to produce a diagnostic socket of a PETG material that was a solid piece with a 4 mm wall thickness (no infill) in 1.5 hours and 15 minutes technical post processing of the socket for patient use. This material was able to be modified during the fitting appointment with the same processes as traditionally fabricated materials and was able to be used in both static and dynamic fittings. Reaching this benchmark enabled our practice to fully adopt a digital workflow. We were able to drop our practitioner's time from 1 hour to 15 minutes and our technician's hands-on time from 1.5 hours to 15 minutes. This was an incredible efficiency booster on both our clinical and technical sides and led to mass adoption in our practice.

## STRENGTH TESTING IN 3D PRINTING

One of the biggest questions we had in the initial implementation of 3D printing prosthetic sockets with High-Flow printers was: Are they equivalent in strength compared to a traditionally fabricated socket? Anecdotally within our clinics, we saw that the strength of the PETG sockets we were producing were similar to that of traditional diagnostic sockets. To explore this further, we partnered with Rosalind Franklin University in North Chicago, Illinois by collaborating on a Department of Defense funded grant, under the Orthotics and Prosthetics Outcomes Research program. We conducted a systematic review to better understand the current state of this newer fabrication method, with a focus on the structural integrity of 3D printed sockets and factors that can affect the strength of 3D printed sockets when tested using ISO 10328 standards. Based on our search, we determined that direct comparison between studies was challenging based on methodological differences including limited sample size, different testing conditions, infill percentages, and reinforcements near the socket pylon interface. Regardless, our systematic review results did show that 3D printed sockets were trending towards producing similar failure forces as those observed in laminated sockets.^5^ We complemented this review with empirical data collected by our group in which we performed ultimate failure testing of 3D printed composite sockets using some of the latest filaments available. For 3D printed sockets, three different material filaments were used: PETG (Filament Innovations, Pennsylvania, USA); polycarbonate or PC (Polymax-PC, Polymaker, Changshu, China); and co-polymer polypropylene or CPX (Filament innovations, Pennsylvania, USA). CPX is a specific co-polymer polypropylene filament that, as per manufacturer information, has higher strength characteristics than standard co-polymer propylene. We refer to the filament using the manufacturers name, CPX, rather than the generic co-polymer propylene to highlight this difference) and compared the results to that of a standard laminated composite (Nano Resin, Paceline Advanced Medical Solution) and a lay-up consisting of braided carbon fiber (ST&G USA Corp.), Nyglass Stockinette (Paceline Advanced Medical Solution), and Nysert (SPS).

In this case, the layup consisted of a layer each of (in this order) carbon braid, nyglass, nysert, nyglass, carbon braid and feather stretch nylon stocking. Another polyvinyl alcohol bag was placed over the layup. The thermosetting resin, activated with the promoter or hardener, was then poured into the bag and was subjected to vacuum to evenly spread the resin throughout the layup on the mold. Manual stringing of the resin was performed from the outside of the PVA bag to ensure the lay-up was fully saturated. The resin started thermosetting shortly after and was left to cure for several hours before it was ready to be cut and trimmed) socket.

The mechanical strength of prosthetic sockets was investigated in accordance with ISO 10328 standards. The loading configurations as specified in ISO 10328 reflects loading that occurs either during heel strike (loading condition I), in which the load axis passes from the anterior side of the proximal leg to the posterior side of the distal leg, or toe-off (loading condition II), in which the load axis passes way towards the anterior side to the distal end of the socket. Condition II is the most commonly used testing condition for ultimate failure of 3D printed sockets since it places the socket in its “worst case scenario”, where failure loads are lower due to higher bending moments generated at the distal end of the socket.

Our failure test results performed at Condition II with a 2-inch pylon at P5 loading level (P5 load level of the ISO standards, which targets patients with a body mass of 100 kg and corresponds to 920 N (settling test), 2013 N (proof test), and 4025 N (ultimate test, upper limit) for the tests) showed 3D printed composite sockets had 32% reduced ultimate failure strength when compared to laminated sockets.

However, they may still be safe to use at the prescribed loading levels. Based on the cumulative results of our testing and literature review, we believe that improving the pylon socket interface and inclined layer printing might help improve the strength to match that of laminate composite sockets.^6^

## OPTIMIZATION OF THE DIGITAL WORKFLOW

The implementation of the full digital workflow within our practice group has been an exciting and challenging task. Our goal was to replicate the traditional process and improve both clinical and technical efficiencies. Our practice is currently utilizing printing for diagnostic sockets (PETG-Filament Innovations), co-poly sockets (CPX - Filament Innovations), and flexible inner sockets (TPU-Matter Hackers).

Our clinical workflow model works as follows for our clinicians:

1. **Shape Capture (15 minutes)**
  - Clinician will cast patient and scan outside of fiberglass cast, as this is simpler than scanning inside of cast, and a uniform fiberglass cast is thin enough to capture limb shape and easily be modified out (75%)
  - Clinician will scan limb directly (15%)
  - Clinician will cast patient and mail cast to fabrication team (10%)
    - We currently utilize structure scanners with an iPad and the Willowood Omega Scan Application (Version 4.2.0) or the Orten Capture Front Camera Scanners (Orten 3D Cam 6.0.1 (588)) on iPhone.
2. **Digital modifications (10 minutes)**
  - Digital technicians modify scan from clinician (85%)
  - Clinicians modify scan and send to digital technicians (15%)
    - We primarily utilize Willowood Omega Software (Version 1.7.2 (1.7.19281.01)) for socket modification. We are currently testing Ossur Design Studio (Version unavailable) and Orten Fly Shape (Version 1.3.1 (588)).
3. **Digital Socket design (10 minutes)**
  - Digital technicians create sockets with desired suspension: Pin lock Lanyard, Suction, Vacuum, or Seal-in
  - Socket is entered into a slicer to create G-code for printing. We currently utilized Odin (Filament Innovations, Version 1.2.2) and Simplify 3d (Version 4.1.2)
    Note: We utilize Meshmixer (Autodesk Version 3.5.474) for all design.
4. **Socket is printed and trimmed by digital technicians (1.5–2.5 hours shape dependent)**
  - We currently utilize Filament Innovations Icarus printers, Kratos Printers, and a PVA med Printer for these prints.

## CONCLUSION

In conclusion, our initiatives have successfully provided clinicians with additional time to dedicate to patient care and improved outcomes. Our ongoing endeavors are directed towards further enhancing patient care through the identification of additional procedural efficiencies. The implementation of diagnostic sockets has streamlined initial care processes and accelerated prescription timelines. Notably, co-poly prints (CPX) have emerged as a reliable solution for prolonged diagnostic socket usage and preparatory devices for new individual with amputation, demonstrating sustained efficacy for up to six months. The integration of flexible prints alongside traditional lamination techniques has not only improved material consistency and reduced waste but has also been seamlessly incorporated into the production of definitive sockets. Through the strategic implementation of automation, our practice has effectively lightened the time burden on clinicians, ultimately contributing to enhanced patient outcomes.

## CALL TO ACTION

Take proactive steps to revolutionize the field of Prosthetics and Orthotics through 3D printing. Embrace this transformative technology to streamline workflows, reduce fabrication time, and ultimately enhance the lives of individuals in need of prosthetic and orthotic devices. Collaborate with technical printer manufacturers, software developers, clinicians, and researchers to expand the capabilities of 3D printing, fostering knowledge sharing and research partnerships. Invest in research and development efforts to overcome material limitations and socket strength challenges, introducing new materials and techniques validated through studies. Establish and disseminate best practices for digital shape capture, design, and printing processes, standardizing workflows for reduced practitioner time and increased consistency across clinics. Prioritize education and training for clinicians, equipping them with the necessary skills for effective 3D printing technology utilization. Maintain a patient-centric approach, directing efforts towards improving clinical outcomes and enriching the patient experience through 3D-printed devices. Together, we can unlock the full potential of 3D printing in Prosthetics and Orthotics.

## DECLARATION OF CONFLICTING INTERESTS

The author is an employee of Bionic Prosthetics and Orthotics Group.

## SOURCES OF SUPPORT

This work was supported by the Department of Defense under the Orthotics and Prosthetics Outcomes Research Program, award number W81XWH2010175.

## AUTHOR SCIENTIFIC BIOGRAPHY

**Figure:**
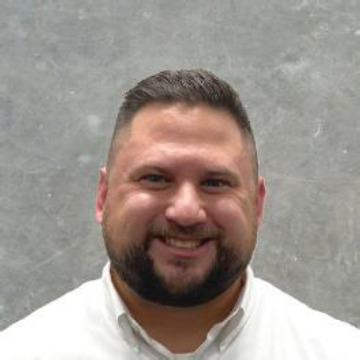


Tony Gutierrez has been with Bionic Prosthetics and Orthotics Group since 2015, currently serving as their National Clinical Specialist for Advanced Prosthetic Solutions. In his role, he leads the group's adoption of digital workflows and 3D printing technology, focusing on enhancing clinical research in socket design, strength testing, and adjustable above-the-knee prosthetic socket fittings. Gutierrez's educational background includes a bachelor's degree in Mathematics Education from Indiana University–Purdue University Indianapolis (IUPUI), a Master's in Biomechanics from the University of Wisconsin, and a Postgraduate Certificate in Prosthetics from The Northwestern University Prosthetics and Orthotics Center (NUPOC). His dedication to the field is evident through past publications in biomechanics, gait mechanics, and prosthetic socket design.
